# Evaluation of tractor driving vibration fatigue based on multiple physiological parameters

**DOI:** 10.1371/journal.pone.0254636

**Published:** 2021-07-14

**Authors:** Ruitao Gao, Huachao Yan, Zhou Yang

**Affiliations:** 1 College of Engineering, South China Agricultural University, Guangzhou, Guangdong, China; 2 Guangdong Provincial Key Laboratory of Conservation and Precision Utilization of Characteristic Agricultural Resources in Mountainous Areas, Jiaying University, Meizhou, China; Tongii University, CHINA

## Abstract

The vibration generated by tractor field operations will seriously affect the comfort and health of the driver. The low frequency vibration generated by the engine and ground excitation is similar to the natural frequency of human organs. Long term operation in this environment will resonate with the organs and affect drivers’ health. To investigate this possibility, in this paper we carried out a collection experiment of human physiological indicators relevant to vibration fatigue. Four physiological signals of surface electromyography, skin electricity, skin temperature, and photoplethysmography signal were collected while the subjects experienced vibration. Several features of physiological signals as well as the law of signal features changing with fatigue are studied. The test results show that with the increase of human fatigue, the overall physiological parameters show the following trends: The median frequency of the human body surface electromyography and the slope of skin surface temperature decreases, the value of skin conductivity and the mean value of the photoplethysmography signal increases. Furthermore, this paper proposes a vibration comfort evaluation method based on multiple physiological parameters of the human body. An artificial neural network model is trained with test samples, and the prediction accuracy rate reaches 88.9%. Finally, the vibration conditions are changed by the shock-absorbing suspension of a tractor, verifying the effectiveness of the physiological signal changing with the vibration of the human body. The established prediction model can also be used to objectively reflect the discomfort of the human body under different working conditions and provide a basis for structural design optimization.

## Introduction

The realization of agricultural modernization is an important condition for China to transform from an agricultural country to an agricultural power. The central link of agricultural modernization is the modernization of agricultural operations, i.e., agricultural mechanization. The level of agricultural mechanization reflects the comprehensive level of a country’s agriculture. Under the promotion of the state, the comprehensive mechanization level of agriculture in China has reached 63.48% [1]. The number of tractors has increased year by year, which has greatly improved agricultural production efficiency and operation quality.

Tractors are essential mechanical equipment for modern agriculture and play an important role in agricultural production. For a long time, agricultural machinery companies have focused on the optimization of equipment and functions, but have ignored the human factor [2]. With ongoing modernization, designers gradually began to pay attention to the factor of driving comfort. However, due to the late start of ergonomics in the agricultural field in China, most manufacturers rarely consider the driving fatigue of tractors.

Compared with ordinary vehicles, the working environment of tractors is poor, and the level of the field is uneven. The vibration generated by the engine and ground excitation is complicated, and the working temperature is high [3]. Therefore, according to the above factors, tractor drivers are prone to psychological fatigue and physical fatigue, which reduces their driving ability. A variety of diseases caused by tractors and work trials often occur, and the health of drivers is seriously endangered [4].

The physical fatigue of the tractor driver directly affects the efficiency and quality of their operation. Therefore, we analyze the physiological characteristics of drivers in different stages of tractor driving fatigue, and study efficient methods for detecting the fatigue state of combine harvesters. This is done in order to effectively identify the fatigue state of the driver, protect the physical and mental health of the driver, and ensure the progress and smoothness of the operation [5]. The result has important practical significance and research value.

Fatigue, as a subjective feeling, is a person’s psychological perception of comfort or the lack thereof. Comfort is a subjective feeling and is difficult to explain with an exact definition. Many scholars give different explanations: Driving fatigue refers to the imbalance of physiological and psychological functions of the driver after driving in a certain environment for a long time. Driving fatigue objectively manifests itself as an inability to meet the expected driving conditions. Fatigue is the most prevalent symptom of the chronic fatigue syndrome (CFS), yet the natural history of fatigue is poorly understood and relatively little attention has been given to its reliable and valid measurement [6]. Fatigue is a universal symptom not only associated with most acute and chronic illnesses, but also with normal, healthy functioning and everyday life [7].

Cao et al. [8] proposed tractor driving vibration fatigue refers to whether the vibration intensity received by the driver’s body is within the range of the human body to withstand, and the degree of fatigue experienced by the human body. Tractor driving vibration fatigue is often evaluated by characteristic indicators, which are divided into subjective indicators and objective indicators [9]. Tian et al. [10] tested the surface EMG signals of the driver’s lumbar erector spinae and multifidus muscles in a simulated tractor vibration environment in 2011. The study showed that the root mean square value increased with the vibration duration, and the average power frequency decreased, combined with subjective fatigue. The survey results showed that the driver feels fatigued after 50–60 min. Ao et al. [11] conducted tests on four different vehicles on five different types of roads, and combined polynomial regression models and subjective scales to get the results. The results show that the driver’s dynamic comfort is the most reliable evaluation of driving fatigue indicator. Lee et al. [12] took the distribution of human sitting pressure and anthropometric values as independent variables, and comprehensive fatigue the dependent variable, and combined with subjective questionnaires and linear models established a seat static comfort evaluation method.

The follow scholars built a model of a tractor’s driver’s seat from the perspective of a man-machine interface. By using CAD, CATIA, etc. to build a non-size model of the human body and match it with the seat model in a software environment, they established the corresponding models of the driving geometric comfort.

Chen et al. [13] used a finite element analysis simulation method in 2017, combined with tractor seat parameters and pressure distribution, to establish a seat static fatigue evaluation method based on H-point device simulation. In 2010, KIM et al. [14] anthropometry of the North American population, human body models were developed for seat comfort simulation, as a practical application of the model in a design process, comfortable driving postures were constructed by adopting the cascade prediction model (CPM), which introduced the detail modeling process of finite element modeling and its results.

Most of the current evaluation methods start with the vibration amplitude and frequency, seat size, and driving conditions to infer the corresponding fatigue status of the human body. In addition, there are few evaluation schemes that consider the real physiological response of the human body. Many rely on subjective feelings for evaluation, and their credibility needs further verification. Therefore, this article starts with objective physiological parameters, which can directly reflect the physiological conditions of the human body, thereby constructing an evaluation system based on human physiological data.

## Materials and methods

During the operation of a tractor, strong vibrations are generated. There are two main sources of vibration, namely, the vibration of the tractor’s power source and the ground excitation. Vibration is transmitted to the human body through the bottom of the cab and the seat. Moreover, the driver’s physiological signals will be affected by many factors such as temperature, humidity, and road surface smoothness which are not conducive to the experimental study of the impact of vibration on human fatigue. Therefore, it was necessary to design a tractor vibration simulation platform to provide a basis for fatigue evaluation test.

### Collection of vibration data of tractor field operations

This study was mainly focused on the fatigue characteristics of tractor drivers, in this section, we discuss the collection of the vibration signal in the cab during the operation of a tractor. But due to the limitations of complex environment and time, this test was carried out in Zengcheng test base of South China Agricultural University in South China region.

**Fig 1:**
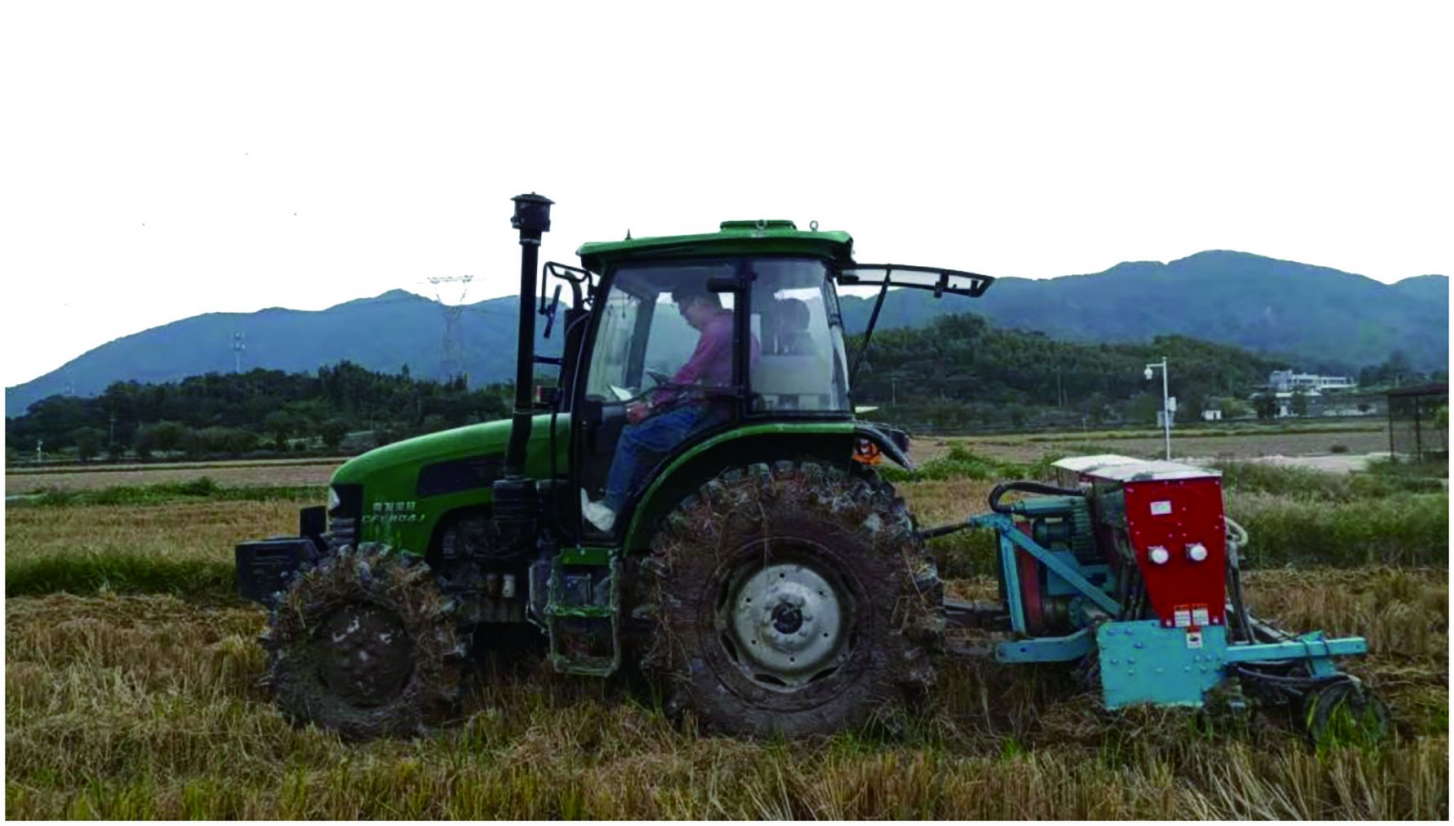
Environment of tractor vibration collection.

The environment conditions were as follows: the altitude was 700–1100 m, the atmospheric pressure in tests area was 1027.6–1028.2 hPa, the temperature was 27–35°C. The environment of tractor vibration acquisition is shown in Fig 1.

The tractor is used for harvesting, sowing, harrowing and weeding by equipped different farming tools. During the test, the tractor drivers are required to complete the task as usual, professional tractor drivers were required to assist in the data collection, and complete the daily task of harvesting, weeding and loosening soil in the process of data collection. This work was carried out in wetland with high soil moisture and less water in surface.

**Fig 2:**
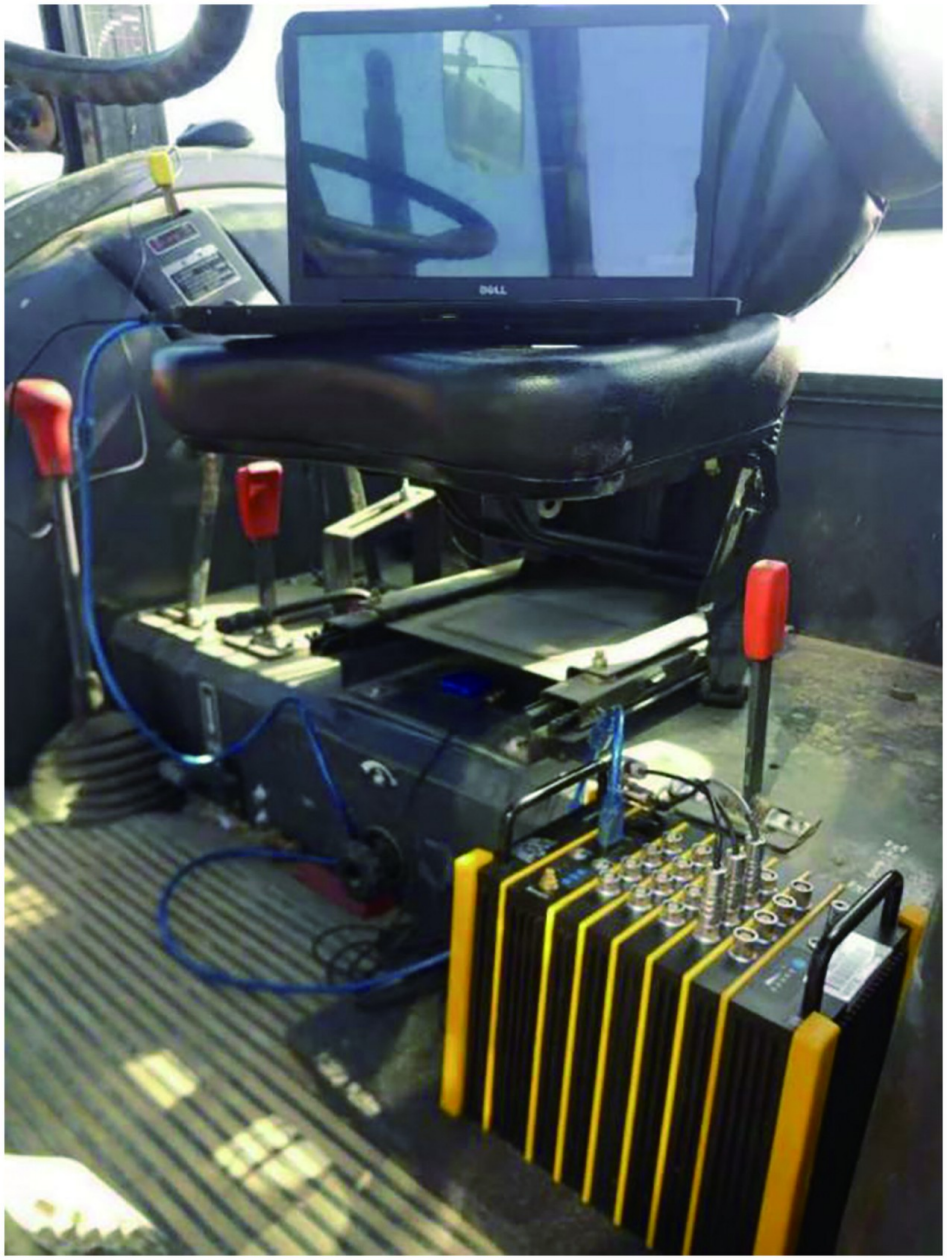
Arrangement of collection equipment.

The tractor model was CFF804Js equipped with working tools at the back. In order to be close the actual state, the three-axis acceleration sensor was installed on the geometric center below tractor seat. Fig 2 shows the data acquisition in the tractor cab, the three-axis sensor was connected to the acquisition module first, and then the data was transmitted to the computer.

**Fig 3:**
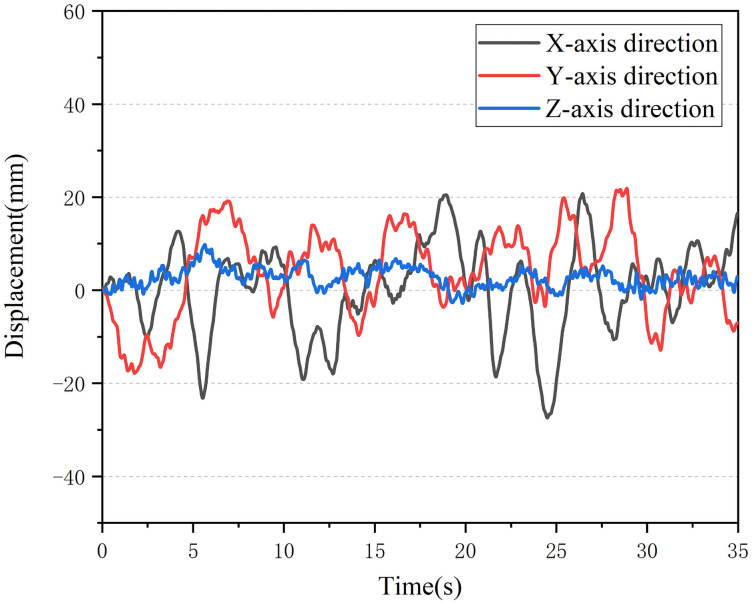
Displacement change curve.

After the acceleration signal was collected, the acceleration signal is integrated twice to obtain the vibration amplitude of the seat. The vibration displacement is shown in Fig 3. In the paddy field environment, the vibration amplitude of the tractor seat chassis is mainly concentrated in 5–25 mm, and the vibration frequency is concentrated in 2–5 Hz.

### Construction of simulation vibration platform

The vibrating platform is composed of a lower base, an upper platform, and six electric cylinders connected in parallel. The electric cylinders are connected with the upper and lower platforms by universal hinges. The six electric cylinders can move linearly in the axial direction of the cylinder. The platform achieves six-degree-of-freedom movement through the coordinated actions of six electric cylinders. The overall parameters of the platform are shown in the Table 1.

**Table 1:** Parameters of vibration platform.

Parameters	Size
Degree of freedom	6
Payload	Max 300kg
Platform size	φ1100mm
Pitch angle	+/-18deg
Roll angle	+/-17deg
Yaw angle	+/-18deg
Vertical lifting Distance	+/-270mm

**Fig 4:**
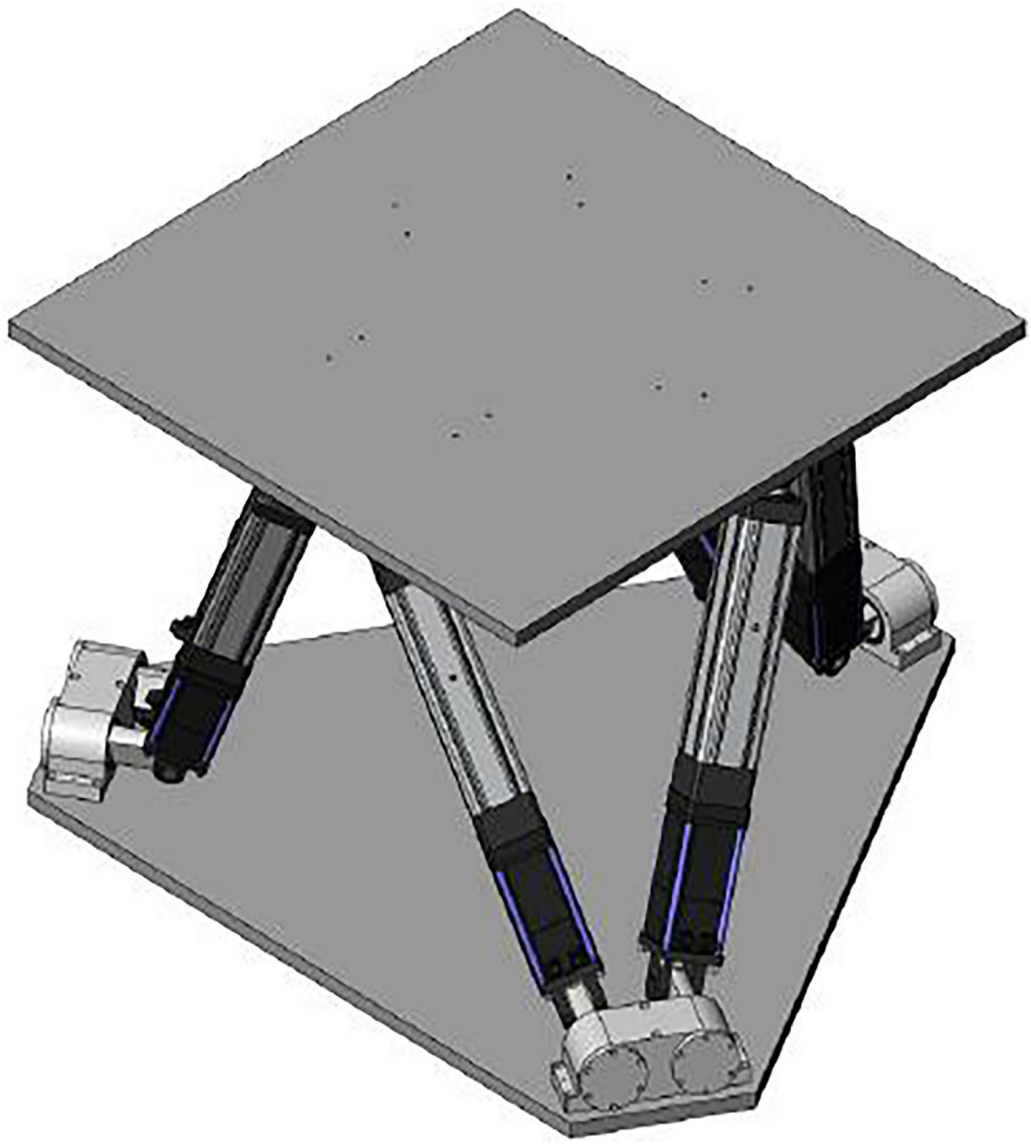
Vibration platform.

The six supports of the parallel mechanism are six electric cylinders. The six-degree-of-freedom motion platform runs under the detection and control of the control system and sends the control signal to the real time control computer. The computer control system calculates the position cylinder length and changes the electric cylinder length by driving the servo motor to realize the motion of the motion platform with six degrees of freedom. The overall structure is shown in Fig 4.

**Fig 5:**
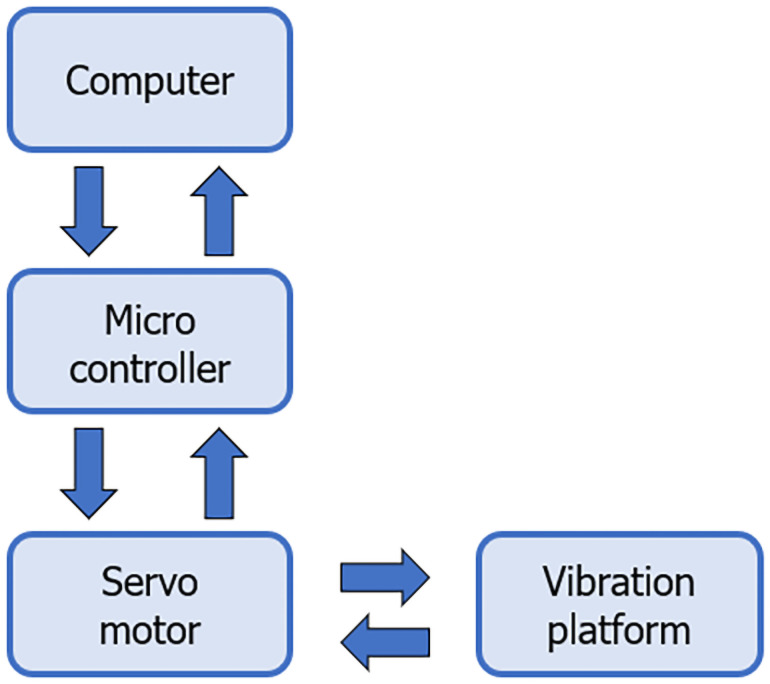
Control flow chart.

The vibrating platform is controlled by a servo system. The control system flow chart is shown in Fig 5. The position command issued by the computer calculates the elongation of the six electric cylinders through the micro-controller inside the vibrating platform and transmits it to the driver. The driver drives the motor to move after receiving the action information. In addition, installing an encoder on the motor allows detection of the torque, speed, and position information of the motor in real time. This information is fed back to the controller, thus forming a closed-loop control system.

### Physiological signal collection under simulated driving vibration environment

The objective method of detecting driving vibration fatigue is to use instruments, equipment, and other auxiliary tools to detect the physiological or psychological specific parameters that can reflect fatigue when the driver is driving [15]. This method is the main development direction of the future research on driving fatigue. There are many types of physiological signals for detecting drivers’ fatigue, mainly including electro-oculogram (EOG), skin surface electromyography (sEMG), electroencephalogram (EEG), respiration (RESP), skin temperature (SKT), photoplethysmography (PPG) and electrodermal activity (EDA). Each kind of signal presents a unique pattern when the body’s metabolic level and fatigue degree change [16].

**Fig 6:**
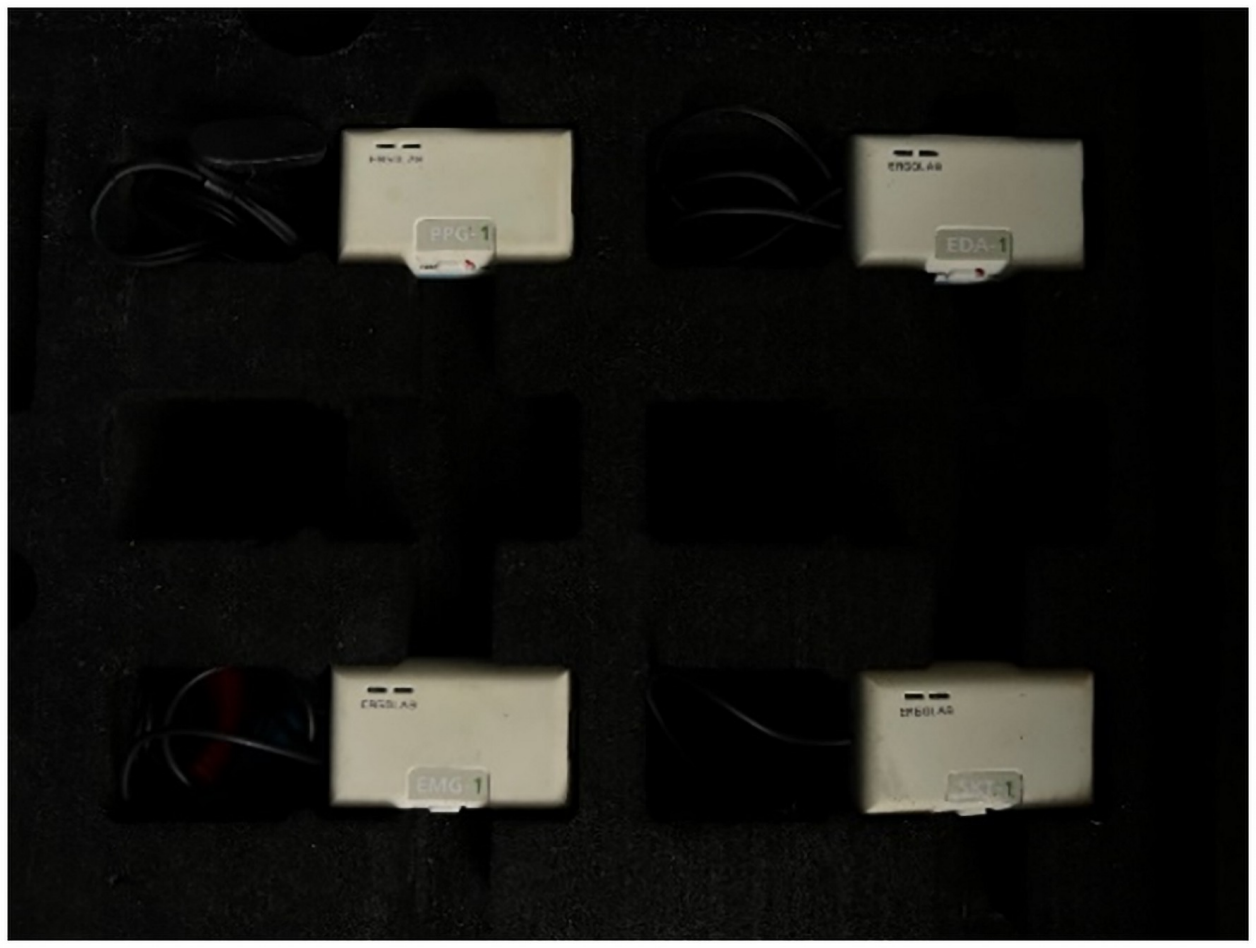
ErgoLAB physiological sensors.

In this simulated tractor driving vibration fatigue test, we input the vibration data under the tractor seat collected in the field work to the vibration platform. As shown in Fig 6, the physiological acquisition module of ErgoLAB was used to collect data from the subjects, which includes the following four physiological signal sensors: EDA, SKT, PPG, and sEMG.

**Fig 7:**
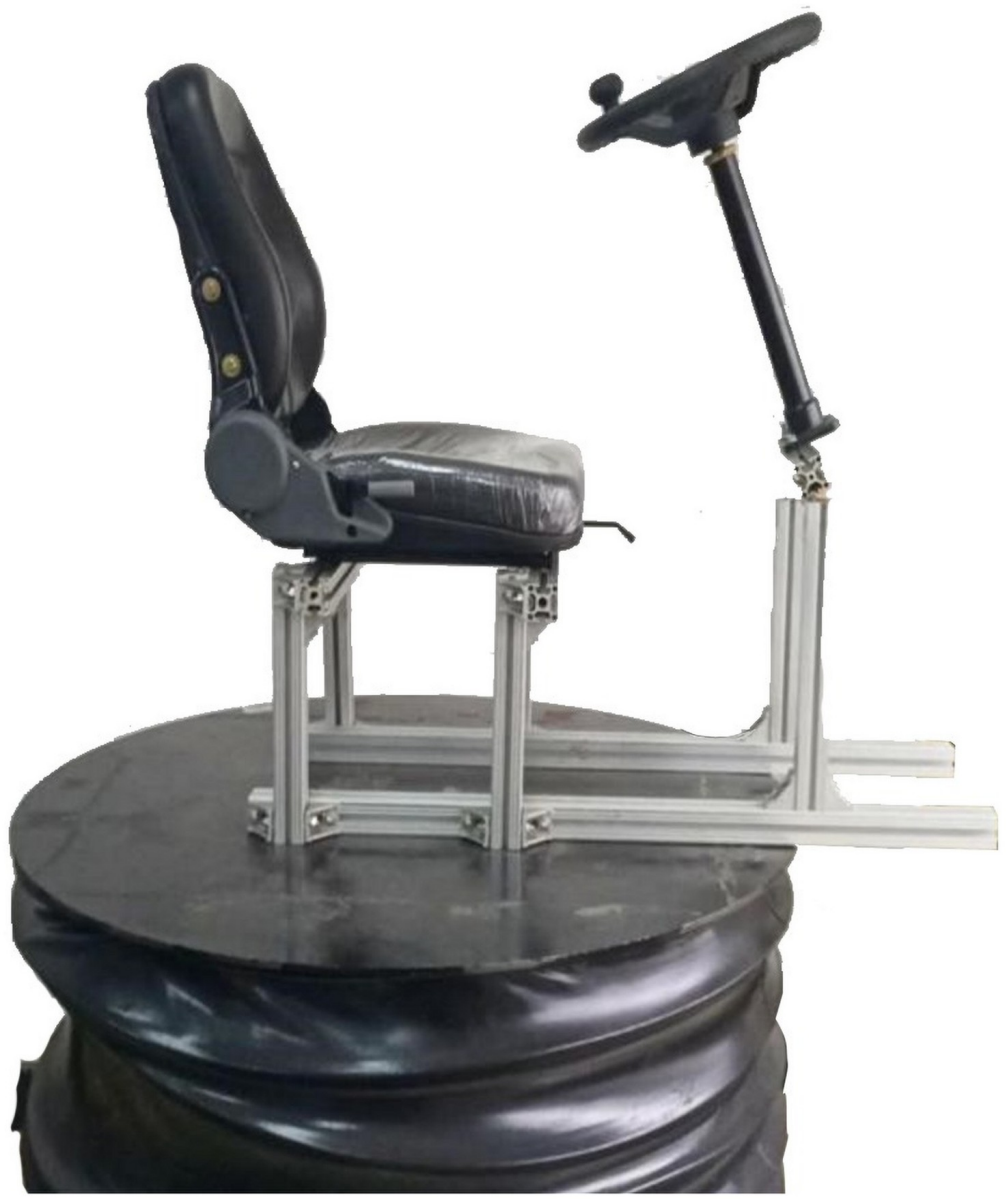
Vibration testing device.

This research was carried out with the approval of the Industrial Design Ethics Committee and the Director of the Industrial Design Department of the College of Engineering, South China Agricultural University. All the subjects were informed of the environmental conditions of the vibration test in advance, and the test was carried out after each subject knew and agreed. The experiment was carried out on the vibration platform previously built which is shown in Fig 7.

In order to strictly control the influence of other factors. Given that people have different levels of fatigue at different times of the day, each test time was uniformly selected after the lunch break every day (14:30–17:30), ten people were selected for the test with driving experience whose BMI values were within the normal range for adult males. Each collection time lasted 120 min.

## Processing and analysis of physiological signal

In the previous section, the number of vibrations under the tractor seat was collected through the field operation of the tractor, and the field collected data was input to the vibration platform to simulate the driving environment. Four physiological signals were measured in the simulation experiment.

**Fig 8:**
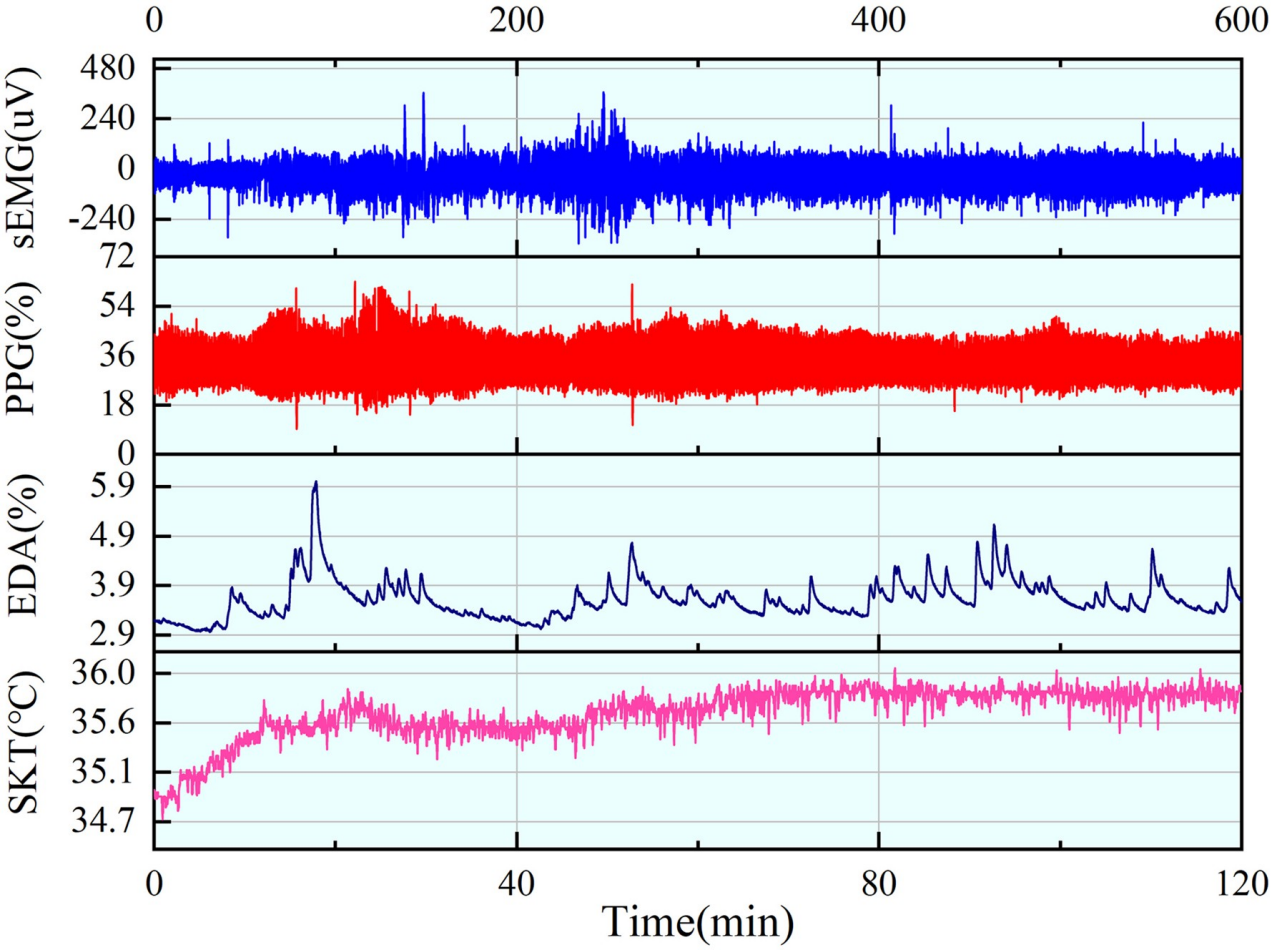
sEMG, PPG, EDA, and SKT signals.

The raw physiological signal usually does not reflect the signal characteristics directly, and it always contains a lot of noise signals, such as interference caused by alternating current and baseline drift caused by small electrode displacement. As a result, noise reduction processing is required for various signals. Therefore, it is necessary to perform noise reduction processing for these signals and retain the accurate characteristics of physiological signals. The physiological signals collected are shown in Fig 8.

First, the complete signal is equally divided into 60 segments. Since the acquisition will take up to 120 min, it is difficult to analyze the relationship of each signal with fatigue as a whole, so it is necessary to separate the signals, further extract the characteristics of each segment, and analyze the underlying pattern of changes with fatigue. In general, the collected signals will be affected by power frequency interference. The following signals are all processed by 50 Hz band-stop filtering, followed by extraction of the parameters of each physiological signal.

### SEMG signal

**Fig 9:**
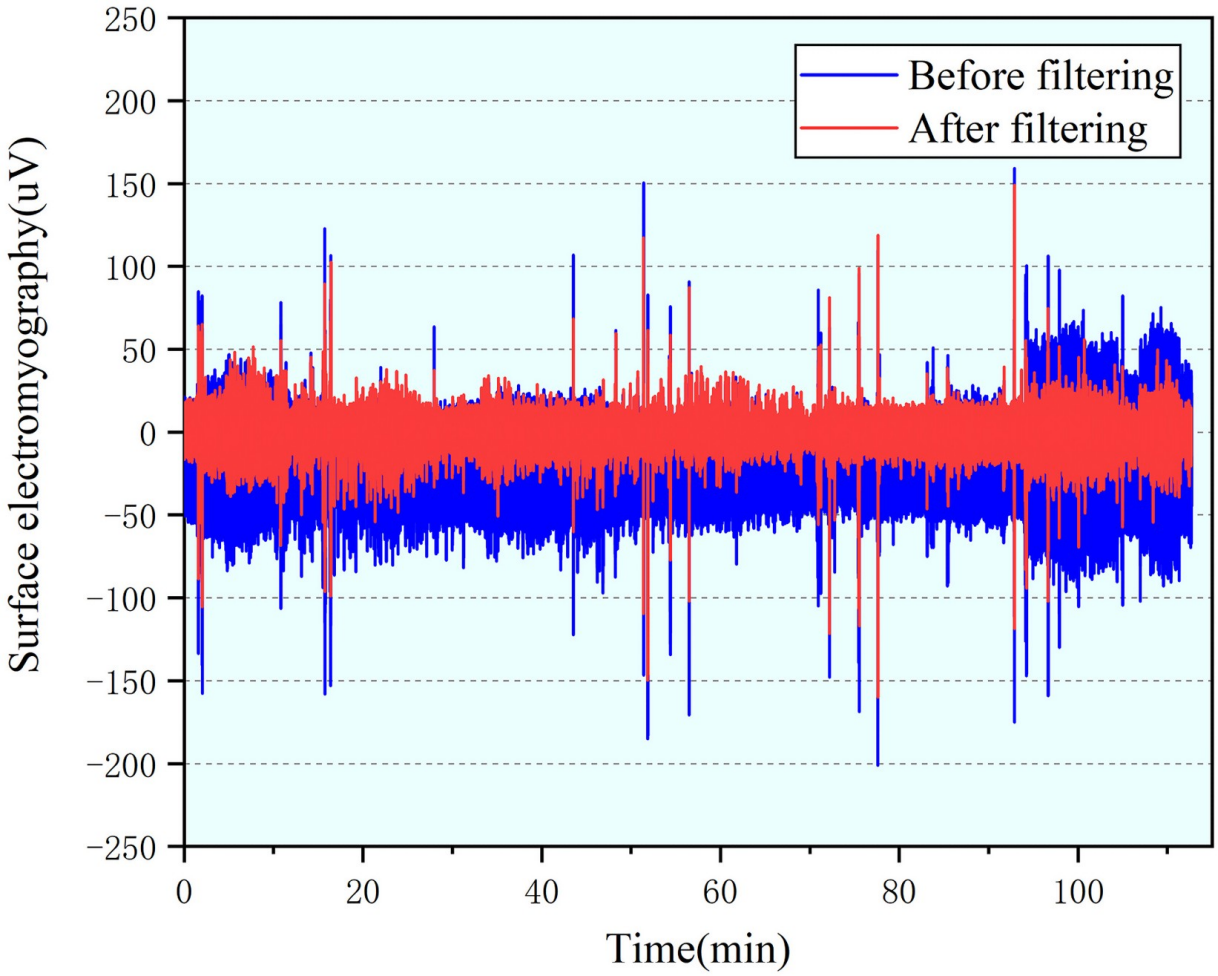
Comparison of sEMG signal before and after filtering.

sEMG is a nonstationary micro-electric signal which will corrupted by many noise The amplitude of sEMG is usually between 0 and 1.0 mV, with the useful signal frequency between 0.1 and 500 Hz, and the main energy concentrated between 20 and 150 Hz [17]. There are many random interference in raw sEMG signal collection, such as the placement of electrode pads, the temperature and humidity of the environment, or the mutual interference of the potential signals of the motor units in the muscle group [18]. Therefore, by using a combination of 0.1 Hz high pass filter and 500 Hz low pass filter to clean sEMG signal while retaining the characteristics of the sEMG. The denoised sEMG signal is shown in Fig 9.

The denoised Subject’ sEMG signal can be used to calculate indice at each time segments. The power spectrum of the sEMG signal will shift to the right when muscles become fatigue where the mean frequency can reflect this change. Mean frequency is defined as:

$$MF\;=\;\frac{\int_{0}^{\frac{f_{s}}{2}}f∙p(f)df}{\int_{0}^{\frac{f_{s}}{2}}p(f)df}$$ (1)

where *p(f)* means the power spectral density of the sEMG signal, and f_s_ is the sampling frequency.

**Fig 10:**
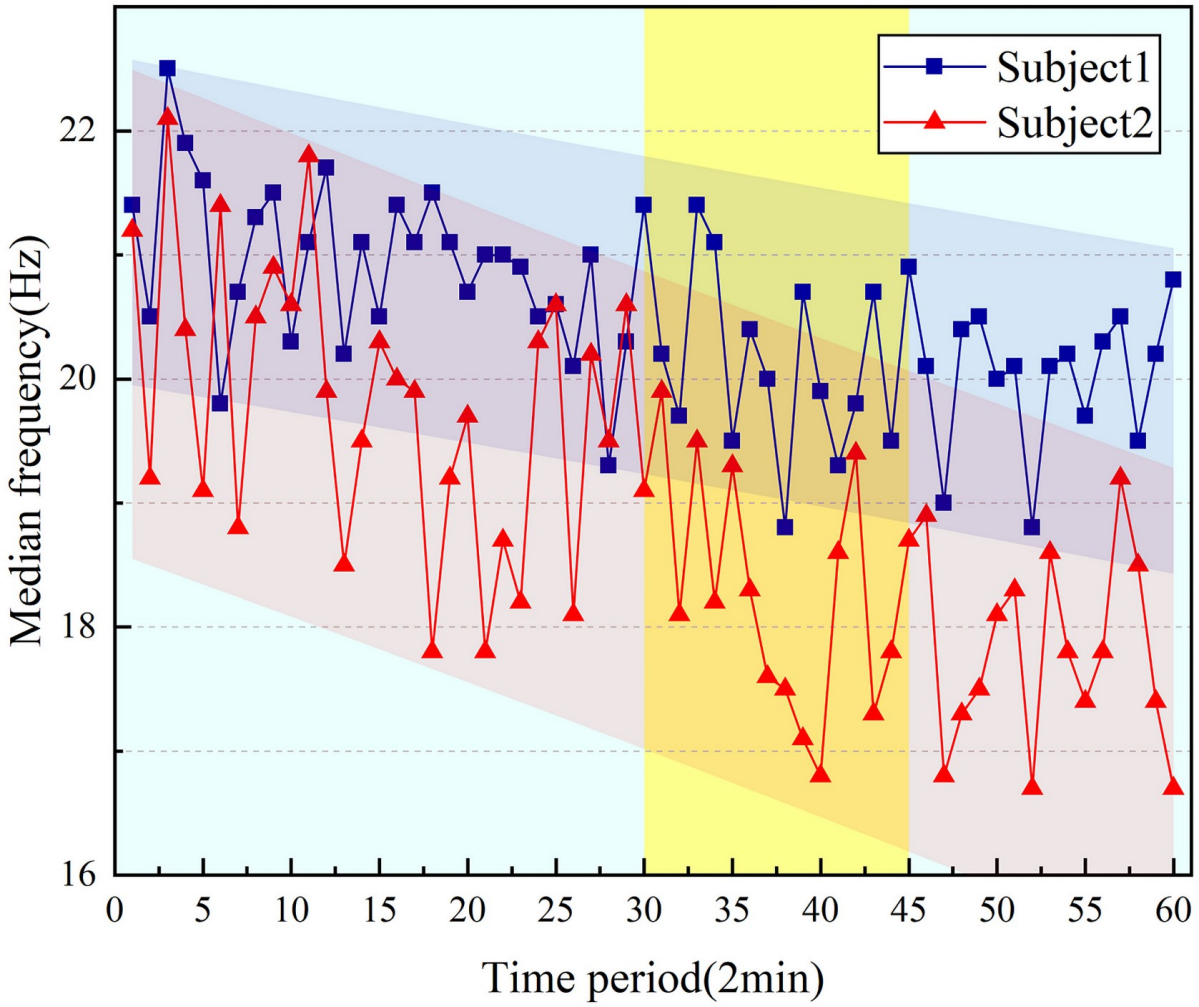
Change of sEMG median frequency.

Two of the ten subjects sEMG signal parameters are selected, as shown in Fig 10. When the driving vibration continues over time, the electromyographic signal MF shows a downward trend. Furthermore, the median frequency changes significantly in the 30–45min period.

As shown in Table 2, the correlation coefficients *r* of MF are 0.824 and 0.815, and the goodness of fit *R*^*2*^ are 0.868 and 0.891, indicating that sEMG parameter is positively correlated with time and keep the higher linear correlation. In the statistical analysis of sEMG signal, the significance level *P* of sEMG parameter both lower than 0.01, which means parameters of two groups have higher statistically significant.

**Table 2:** Regression analysis of EMG signal parameters.

Parameters	Y_MF1	Y_MF2
**Intercept**	20.577± 0.251	21.287 ± 0.168
**Slope**	-0.054 ± 0.007	-0.026 ± 0.004
**r**	0.824	0.815
**R**^**2**^	0.868	0.891
**P**	0.002	0.007

### SKT signal

Skin temperature is an important parameter for calculating human energy metabolism and analyzing human body fatigue and other physiological reactions [19]. Under different physiological conditions, the skin temperature relating to different degrees of fatigue will also change regularly [20].

**Fig 11:**
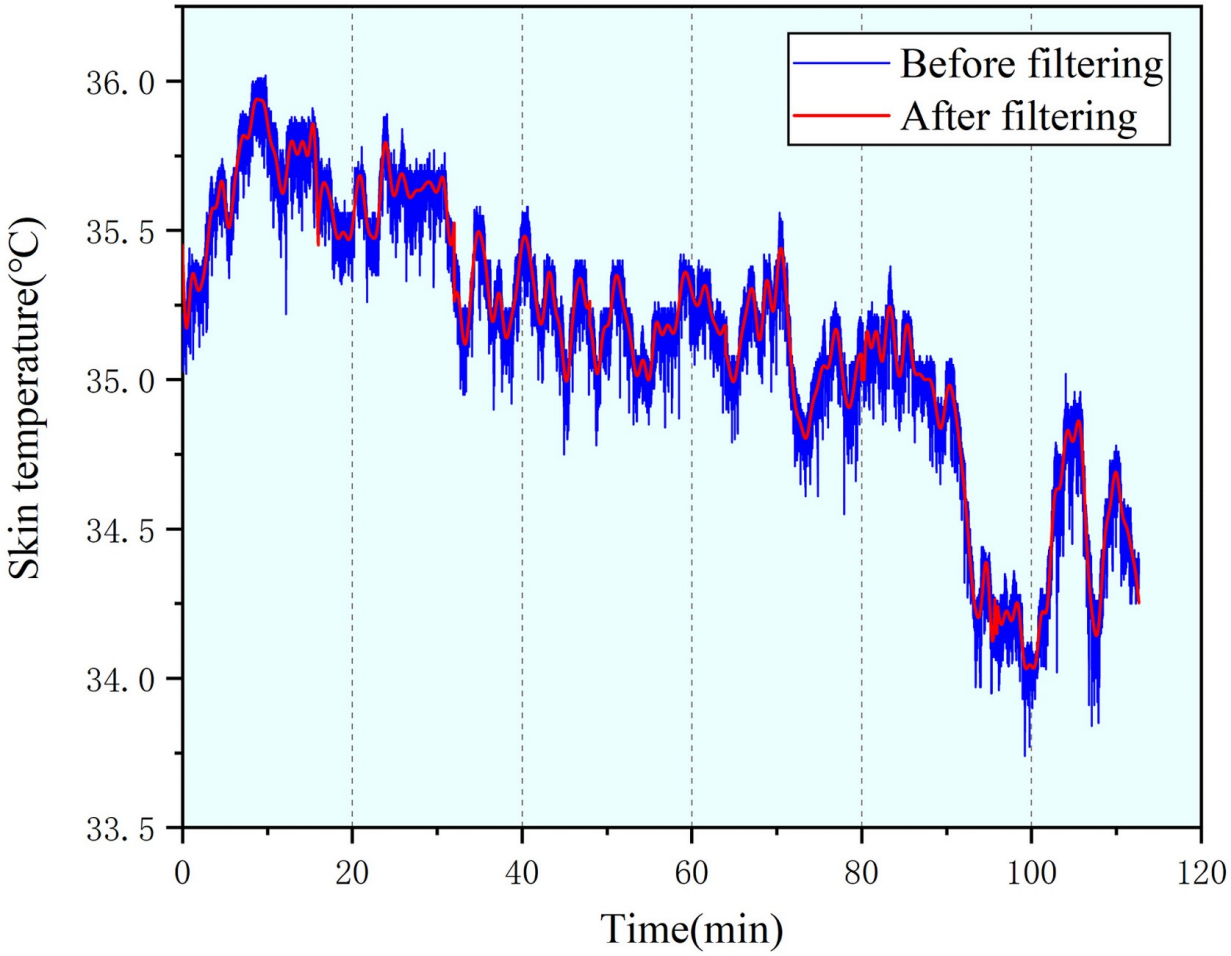
Comparison of SKT signal before and after filtering.

On the basis of filtering the interference caused by the power frequency noise, a 0.05 Hz lowpass filter and a moving average filter with a sliding window of 125 ms are used to remove other interference. The filtering result is shown in Fig 11.

We fit the skin temperature curve of each period by the least square method of first order linear regression, and the calculation method is as follows:

y=α+βx+ε (2)

**Fig 12:**
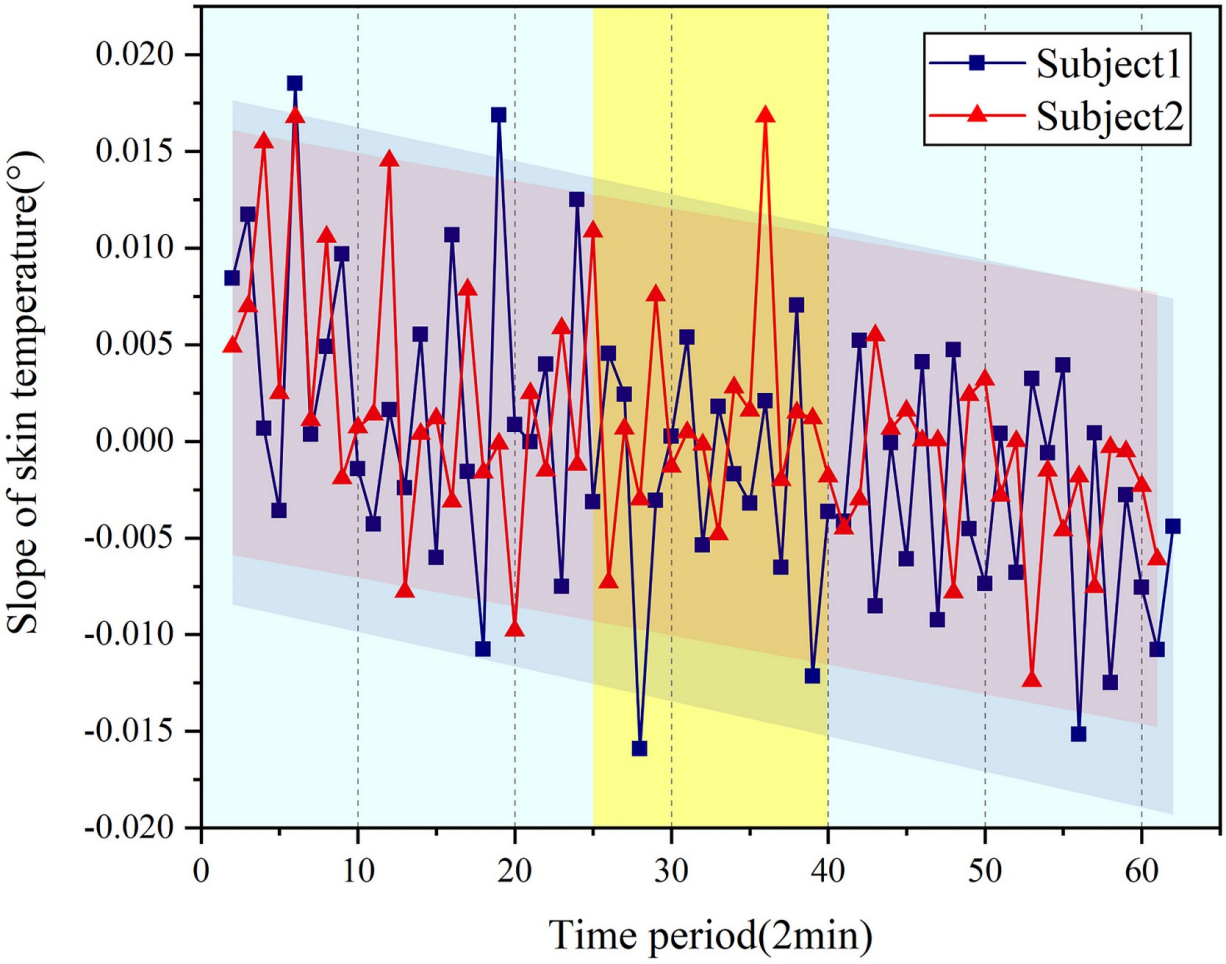
Trend of skin temperature slope.

Where *α* is initial skin temperature, *β* is slope of the each segment and *ε* is the random white noise. The slope of each segment after fitting was extracted. We randomly selected the skin temperature parameters of two subjects from ten subjects, as shown in the Fig 12. In the process of extracting the slope of each segment of the skin temperature, we found that the change speed of skin temperature increases with the increase of the vibration time in the 25–40 time period. The slope of the overall signal curve reflects a process of continuous decrease. The decrease of the fitting slope indicates the increase of driving vibration fatigue.

The fitting characteristics of the two sets of skin temperature parameters are shown in Table 3. The significant differences between the two sets of data P are 0.01<P <0.05, indicating statistical significance. Furthermore, the correlation coefficients are 0.821 and 0.844, which denote the effectiveness in this line fitting.

**Table 3:** Regression analysis of SKT signal parameter correlation.

Parameters	Y_Slope1	Y_Slope2
**Intercept**	0.0054±0.00148	0.0050 ± 0.00175
**Slope**	1.334±0.0014	1.76 ± 0.00047
**r**	0.821	0.844
**R**^**2**^	0.775	0.71
**P**	0.022	0.038

### EDA signal

Electrodermal activity is a characteristic of the human body, which causes continuous changes in the electrical characteristics of the skin. The application of EDA is very extensive, and it is often used in psychological research and human stress response research [21].

According to the signal characteristics of EDA, it can be divided into two categories, skin conductance level (SCL) and skin conductance response (SCR). SCL is the conductance level, which refers to the basic value (μS) between two points on the skin surface when a person is at rest. When the individual is excited, the conductance level is relatively high, and when they are relaxed, the conductance level is relatively low; it shows a trend of continuous fluctuation. The skin conductance response (SCR) is an excited state caused by stimuli, which is manifested as multiple peaks [22].

**Fig 13:**
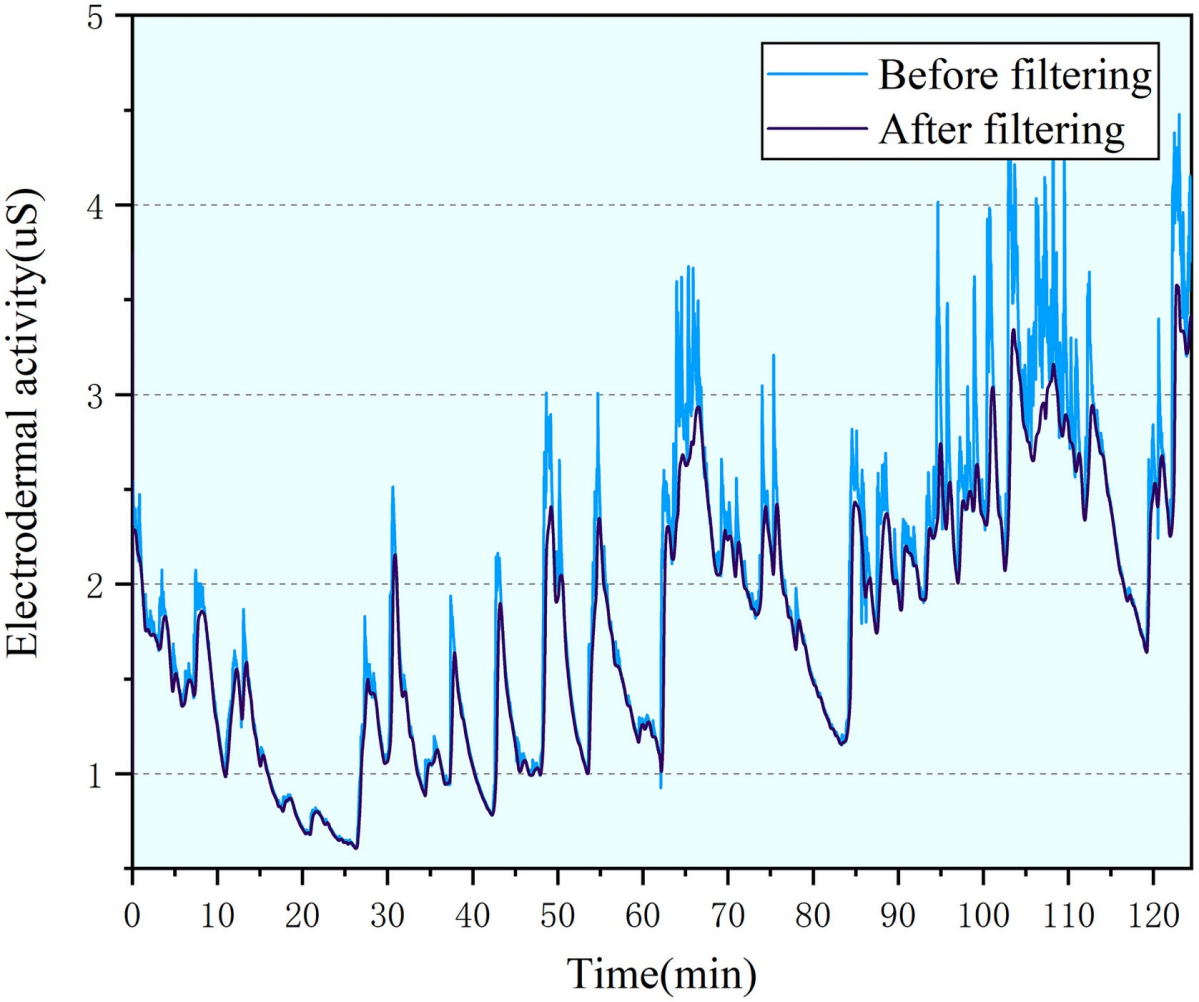
Comparison of EDA signal before and after filtering.

The frequency range of the EDA signal is mainly concentrated below 0.2 Hz, and its effective range is 0.02–0.2 Hz [23]. Due to the influence of machine noise and other external factors, the collected EDA signal is much higher than 0.2 Hz. We used a 0.02 Hz high pass filter and a 0.2 Hz low pass filter and smoothing filter to reduce noise. The filtered EDA signal is shown in Fig 13.

**Fig 14:**
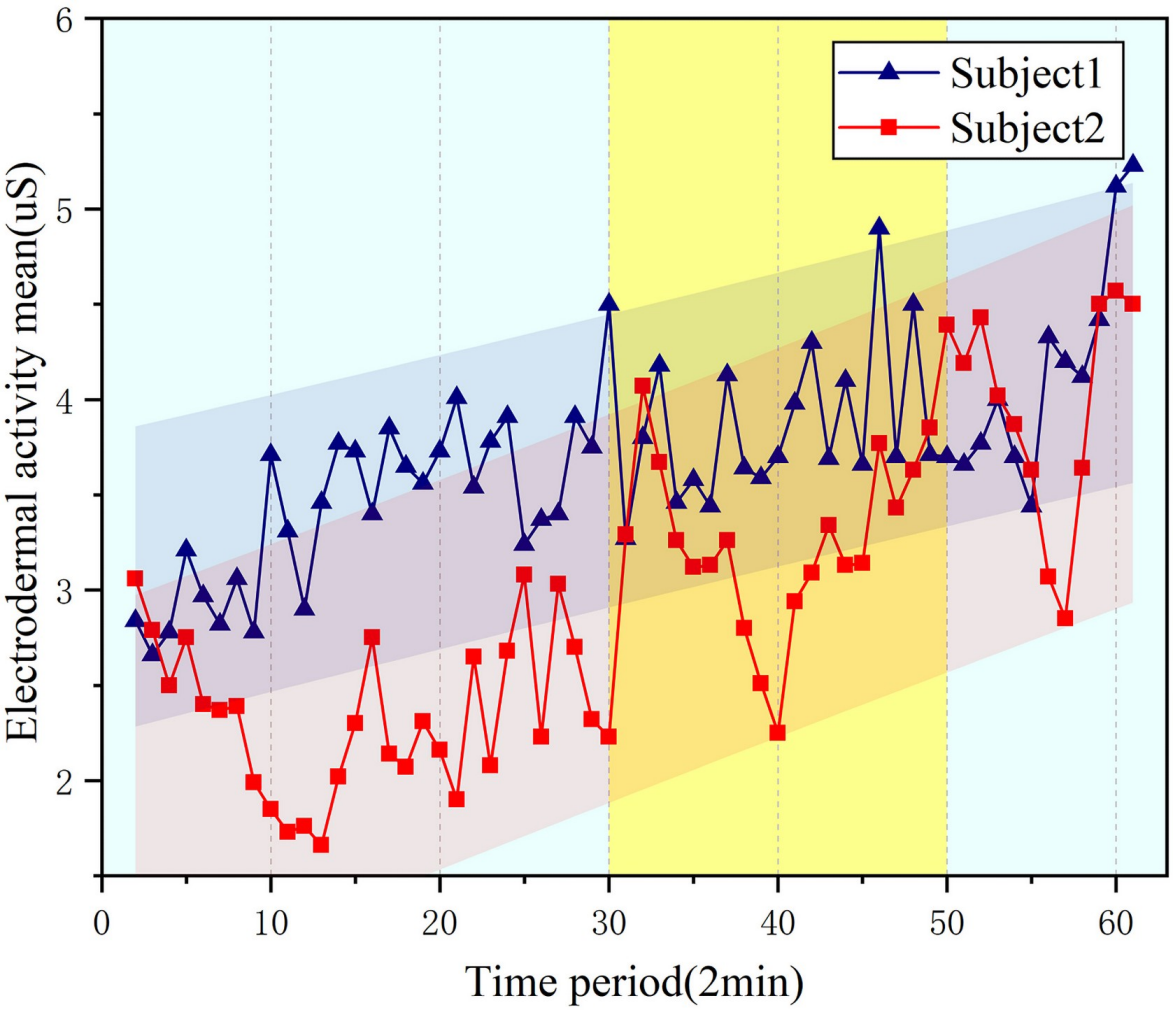
Trend of mean value of EDA signal.

The changing trend of EDA signal parameters can be clearly seen from Fig 14 and the mean value of EDA signal is shown in formula (3), in the 30–45 time period, the mean value of the skin signal is significantly different from other periods, which is manifested as an increase in the amplitude change, but the change of EDA parameters in the initial and final stages maintains a slow increase.


$${\overset{-}{X}}_{mean}=\frac{1}{N}\sum_{i=1}^{N}X_{i}$$ (3)

Where *N* is length of eda signal, and *X*_*i*_ is amplitude.

In denoised EDA signal, by means of filter, noise is eliminated. The signal is analyzed and fitted by computer for statistics, the results are shown in Table 4. The correlation coefficient r of two groups is 0.704 and 0.768 respectively and the goodness of liner R^2^ is 0.821 and 0.844 which prove the regression liner fitted the EDA parameters accurately. In statistical analysis, P value of two groups of parameters both between 0.01 and 0.05 showed the statistically significant of EDA parameters.

**Table 4:** Regression analysis of EDA signal parameter correlation.

Parameters	Y_mean1	Y_-mean 2
**Intercept**	3.028 ±0.103	1.861 ±0.137
**Slope**	0.022± 0.002	0.034 ± 0.004
**r**	0.704	0.768
**R**^**2**^	0.821	0.844
**P**	0.024	0.037

### PPG signal

**Fig 15:**
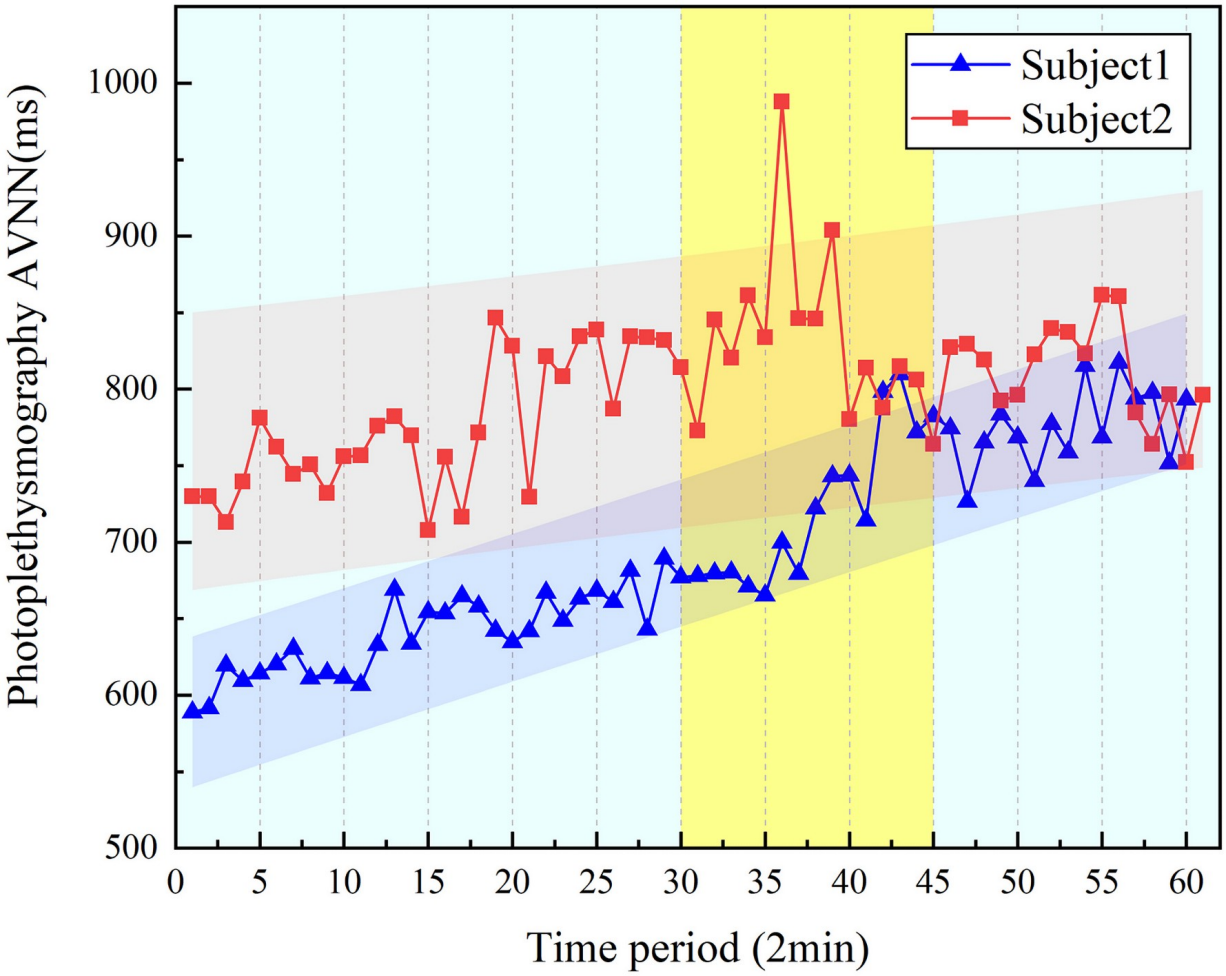
Comparison of PPG signal before and after filtering.

Photoplethysmography signal is a low frequency signal, and the main energy of a normal photoplethysmography signal is distributed in the range of 0.5–5 Hz [24]. The PPG signal reflects the pulsatile changes of blood in the blood vessels during the heart beat cycle which is also a non-invasive detection method by photoelectric means. Heart rate variability (HRV) is currently the main method of analyzing PPG signals and HRV is the fluctuation in the time intervals between adjacent heartbeats. Characteristics of the heartbeats can be reflected in 0.1 to 20 Hz in frequency domain Therefore, we filter out interference by using a high pass filter with a cutoff frequency of 0.1 Hz and a low pass filter with a cutoff frequency of 20 Hz, and filter out the baseline drift of 0.1 Hz. Then, the EMG interference between 0.1 to 20 Hz is removed by wavelet denoising we remove the EMG interference between 0.1 and 20 Hz by wavelet denoising. The filtered PPG signal is shown in Fig 15.

The R-R interval mean (AVNN) is the “gold standard” for medical stratification of cardiac risk [25]. We select the mean value of the original signal cardiac interval (R-R interval) as the characteristic parameter. AVNN can respond to the change of the heartbeat cycle difference from time to time. The AVNN calculation method is as follows:

$$AVNN\;=\;\frac{1}{N}\sum_{i\;=\;1}^{N}RR_{i}$$ (4)

where *N* is the number of heartbeats, and *RR*_*i*_ is the *i-th R-R* interval.

**Fig 16:**
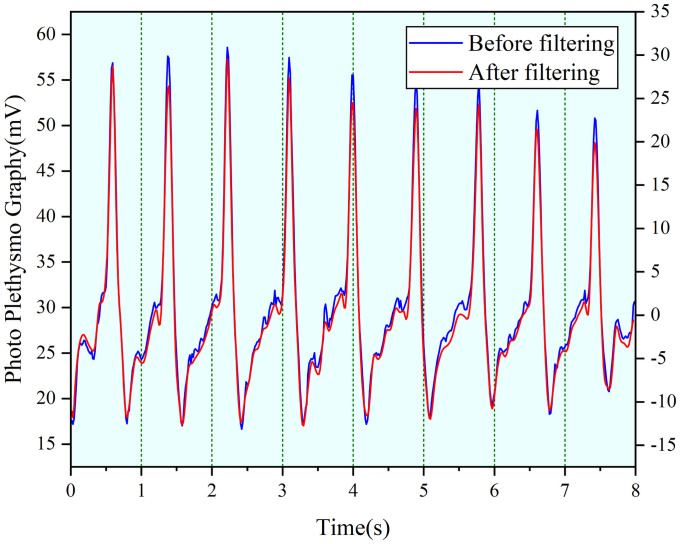
Regression analysis of PPG signal parameter correlation.

We randomly selected the parameters of the PPG signal of two subjects from ten subjects. The changing trend of parameters is shown in Fig 16. It is found that the change of AVNN in the 30–45 min segment increases significantly compared to other periods. As the subjects are exposed to vibration for an increased time, the entire AVNN curve shows an upward trend.

The linear regression analysis of PPG signal parameter is shown in Table 5, the correlation coefficients r is 0.834 and 0.916, and the goodness of fit R^2^ are 0.871 and 0.834, indicating the parameter is positively correlated with the time. The significant difference P values of the PPG parameters are both less than 0.05, according to statistical analysis of parameter, the results show the regression equation have statistical significance and remarkably correlative to the PPG parameters.

**Table 5:** Regression analysis of PPG signal parameter correlation.

Parameters	Y_AVNN1	Y_AVNN2
**Intercept**	789.08±11.504	585.523±6.294
**Slope**	1.334±0.325	3.576±0.179
**r**	0.834	0.916
**R**^**2**^	0.871	0.834
**P**	0.001	0.037

### Subjective questionnaire

In order to accurately consider the situation of fatigue when the human body is exposed to the driving vibration environment, a questionnaire on subjective fatigue was also set up at the same time as the driving vibration test. The questionnaire was set up based on the Likert 5-level psychological scale method, which divides the subjective comfort of the human body into 5 different levels [26]. The division of vibration discomfort level is shown in Table 6.

**Table 6:** Fatigue grading and scoring.

Degree	Level
**No fatigue**	1
**Slight fatigue**	2
**Fatigue**	3
**Relative fatigue**	4
**Extreme fatigue**	5

**Fig 17:**
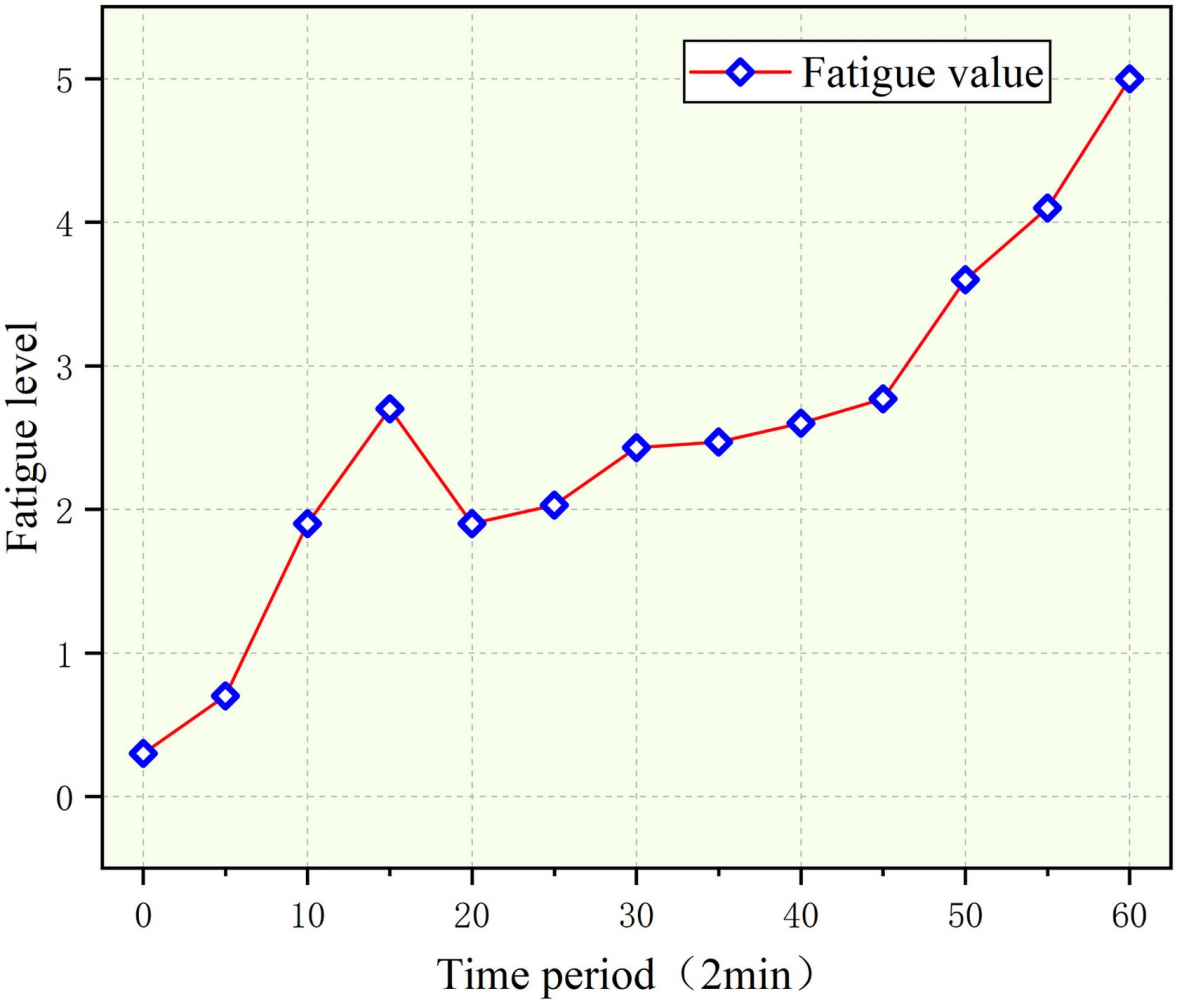
Trend for subjective comfort under vibration.

Under the driving vibration condition of the simulated tractor, the overall fatigue level of the human body shows an upward trend, but the difference between the front, middle, and later stages is quite large which is represented in Fig 17 below. First, in the early stage 0–20 time period, the fatigue level gradually increased to level three; but in the 20–45 time period, the fatigue level first dropped to level 1, and then slightly increased. Next, the fatigue level increased extremely at the 35–45 time period stage and reached the fourth level of fatigue. In addition, when extracting the parameters of various physiological signals, it was also found that the parameter changes in the midterm were significantly intensified, which also verified the characteristics of the mid-term fatigue changes. In the final stage, the fatigue level changes slightly slowed down, and finally reached the fifth level of fatigue.

Summarizing the above data, we came to the following conclusion: the average value of EDA and the AVNN of the PPG signal increases as the driving vibration increases. On the contrary, the median frequency of sEMG decreases and the slope of the SKT signal decreases with increasing driving vibration fatigue. In the middle of the test, the changes in the parameters of the physiological signals increased, and it was also verified that the fatigue level increased significantly in the middle of the test.

Combining the results of the subjective questionnaire survey and the change law of the characteristics of various physiological parameters, it is found that the change law of the physiological signal is highly consistent with subjective fatigue. Therefore, the collected signals can be added to categories according to five fatigue levels.

## Establishment of artificial neural network and verification of fatigue

At present, many studies claim that the level of fatigue changes linearly with time, and the method of time series is used to predict the trend of the change of human fatigue. However, many physiological signals of the human body are nonsequential and nonstationary signals [27]. Therefore, it cannot be simply considered that the change of human body fatigue changes linearly at any time.

### Establishment and training of artificial neural network

**Fig 18:**
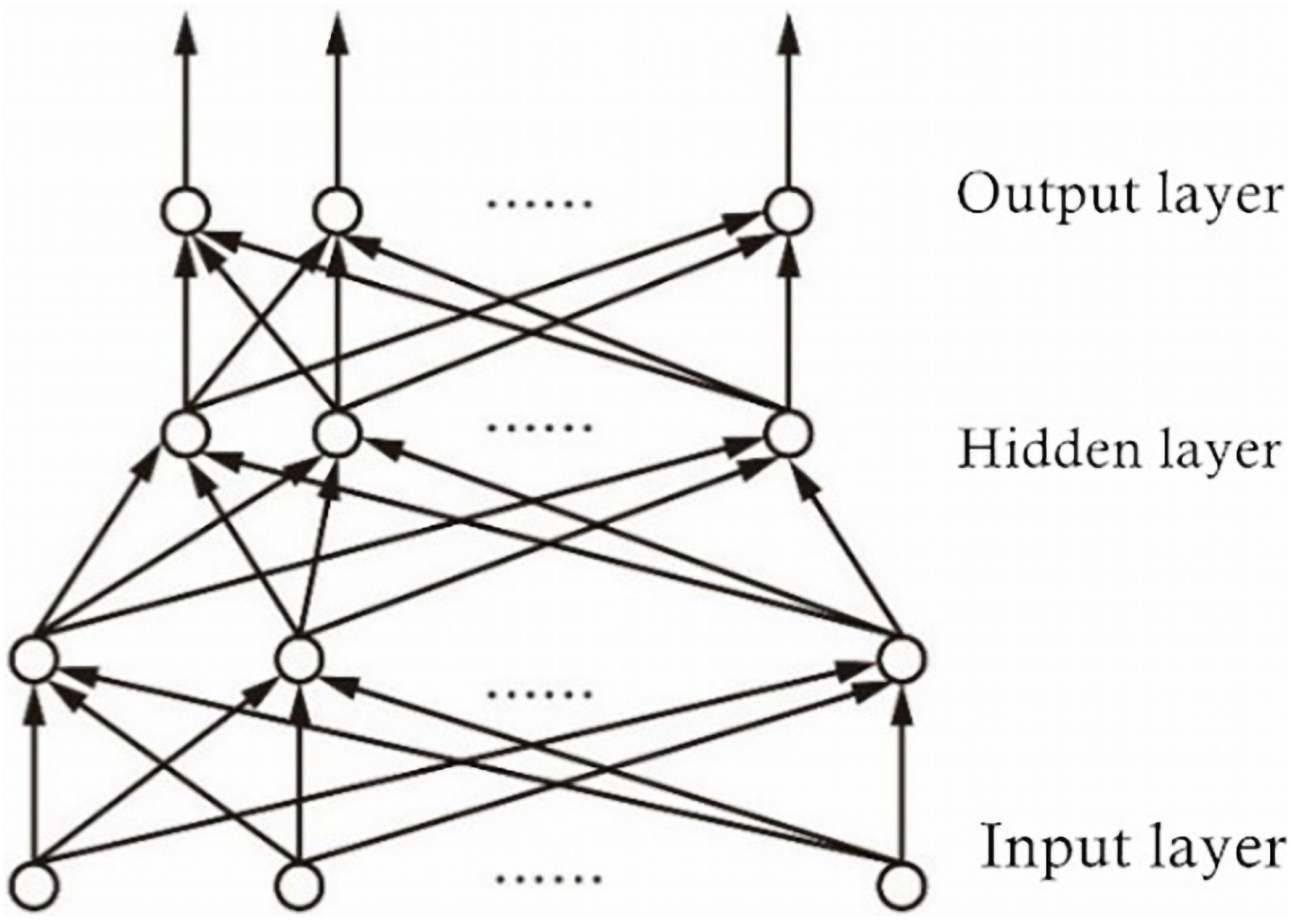
Neural network model.

The nonlinear techniques that are used to estimate body fatigue are based on neural networks to relate the above parameters to body fatigue [28]. The advantage of these techniques compared to the linear methods is that the neural networks can learn the associations between parameters and body fatigue. It has been established that Artificial Neural Network (ANN)-based models closely emulate the decision-making paradigm used by the human brain [29]. ANNs have been a preferred classifier in applications where the training features exhibit nonlinear nature and the decision boundary is best modeled as a nonlinear function in the feature space. Neural network training involves selection of (a) the input, hidden, and output layers in a particular architecture, (b) a learning method, (c) training method, and (d) a stopping criterion. For the current work, we use artificial neural network models, which are used for time series, prediction, and classification domain analysis. The specific structure of the artificial neural network is shown in Fig 18.

An artificial neural network consists of a large number of interconnected neurons (nodes). Each neuron represents a specific output function, called the activation function. The connection between two neurons represents the weighted value of the signal passing through the connection, called the weight. The output depends on the network structure, connection method, weight, and activation function. The mathematical model of ANN is shown in formula (5).


$$N\;=\;f(\sum w_{ij}x_{i})-\theta$$ (5)

Where *x*_*i*_ is the input, *w*_*ij*_ is the weight, *θ*_*i*_ is the interference function, and *f(x)* is the activation function.

**Fig 19:**
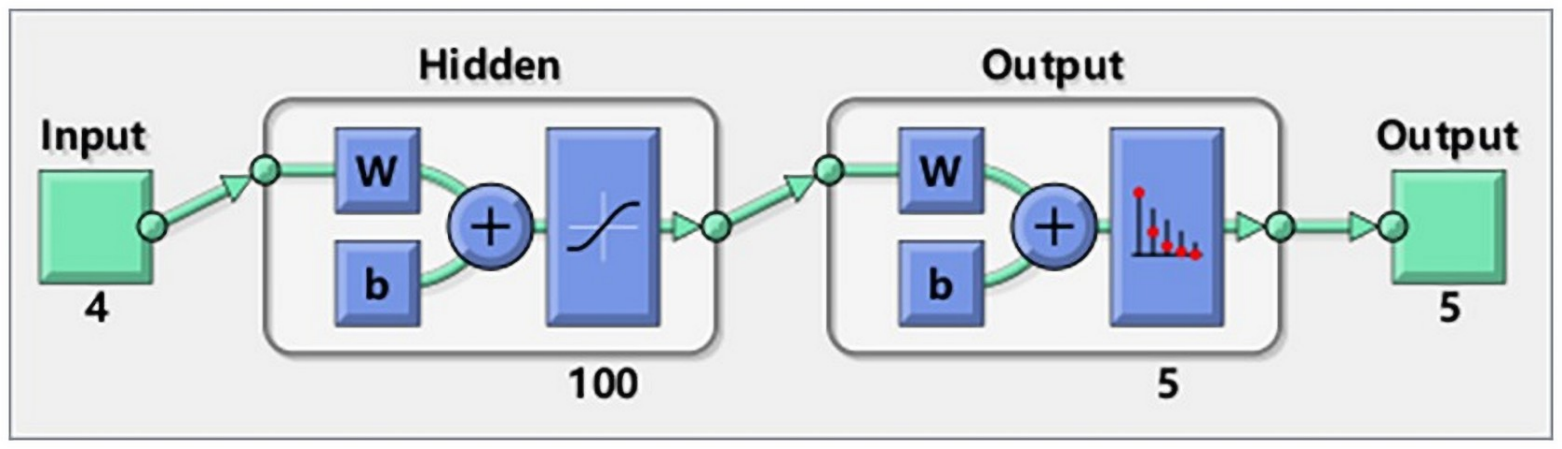
Neural network model of tractor driving vibration fatigue.

In this paper, we constructed an artificial neural network model using Matlab 2019a. Ten people participated in the experiment and the parameters of the four signals are used as input of artificial neural network Concretely, input contains the MF of sEMG signal, the slope of SKT signal, the mean value of EDA signal and AVNN of PPG signal. Minimum input unit was the parameters of each segment for two minutes and whole experiment obtained a total of 2400 samples. The input layer corresponds to four physiological parameters, the number in the output layer represents that the number of output fatigue categories was five, and 100 neurons were built in the hidden layer. The internal structure is shown in Fig 19.

### Results and verification of driving vibration fatigue evaluation

**Fig 20:**
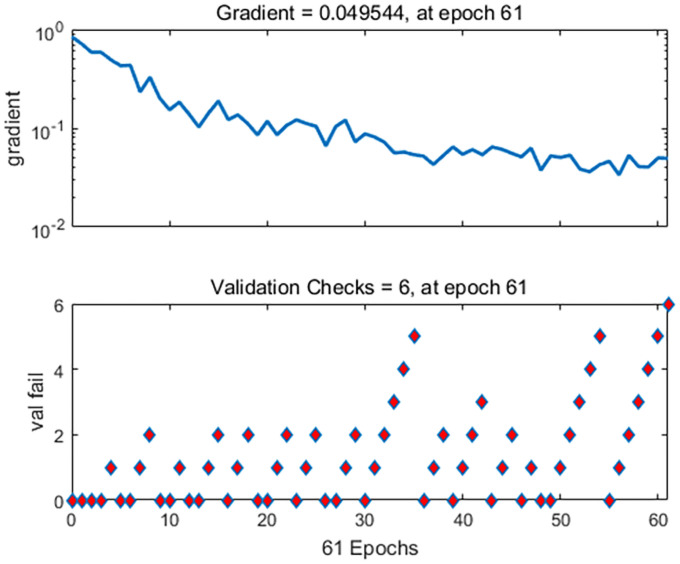
Gradient descent graph.

**Fig 21:**
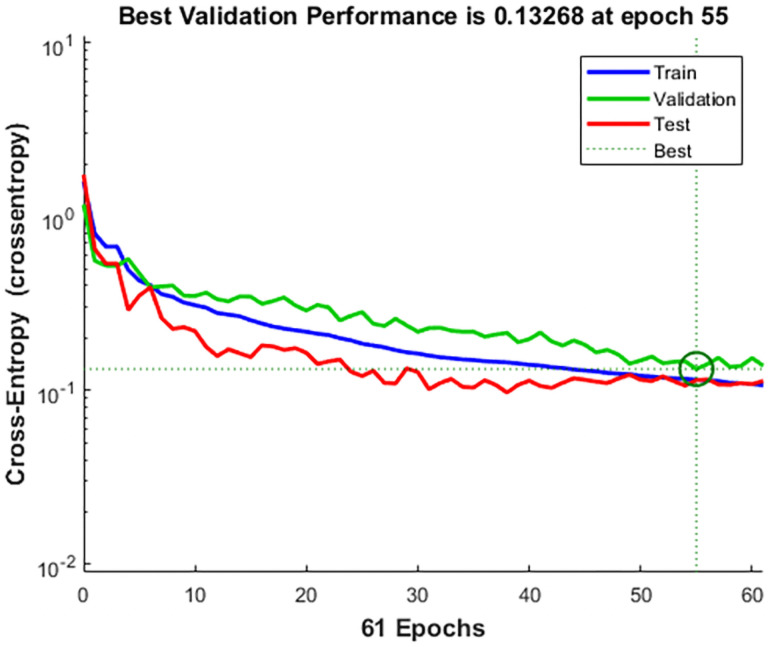
Loss function descent graph.

In the experiment of collecting physiological parameters, a total of 2400 data sets were collected, of which 1680 were used for training, 360 were used for verification, and 360 were used for testing. When the number of iterations reached 60, the gradient value reached a minimum of 0.0495. The gradient change in the model training process is shown in Fig 20, the loss function changes of the training set, test set, and validation set are shown in Fig 21.

**Fig 22:**
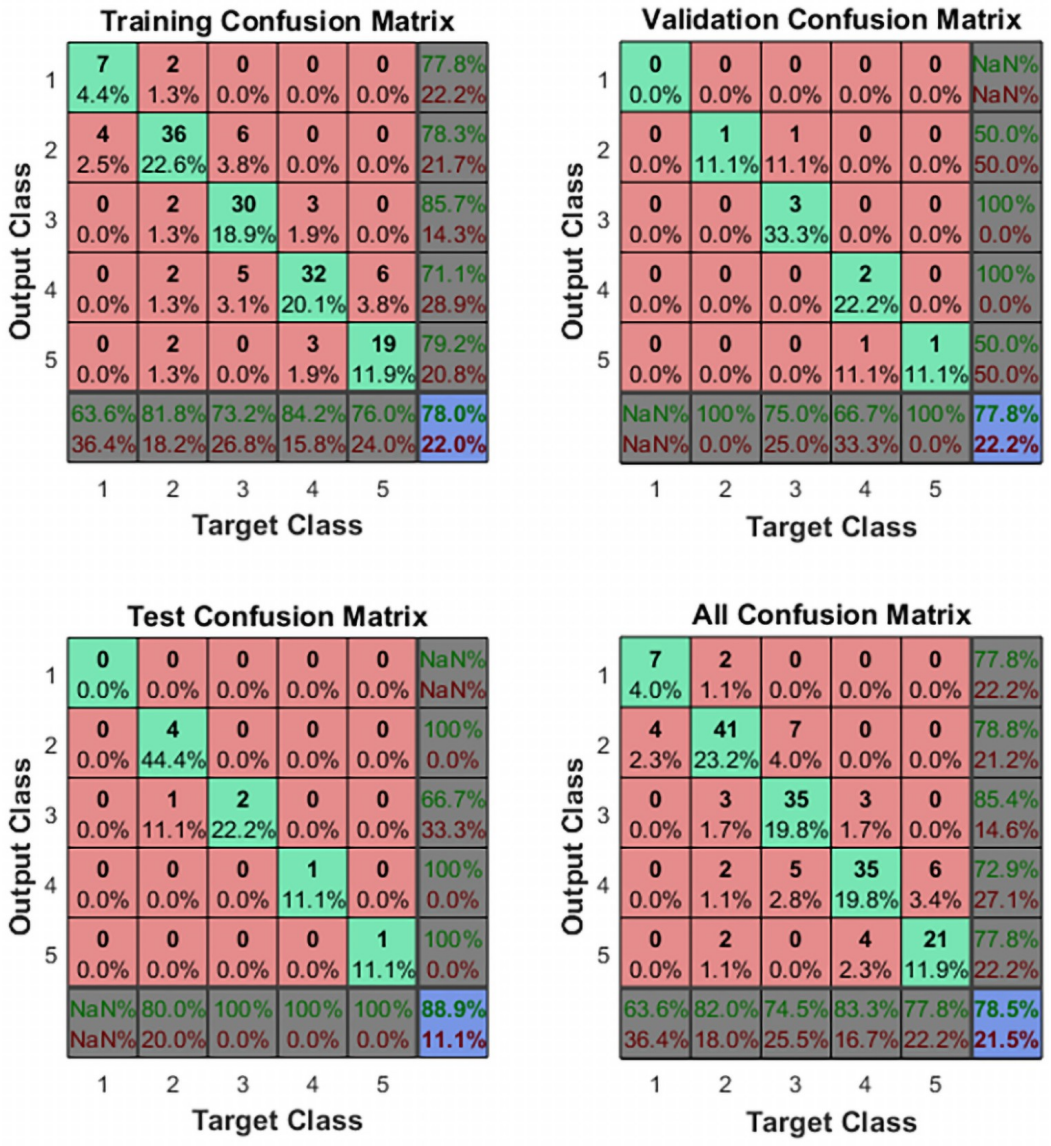
Results of artificial neural networks calculation.

It can be seen from Figs 20 and 21 that the gradient value and loss function reach the optimal value in 55 iterations of the three data sets. This verifies that the artificial neural network has reached the optimal solution here. It also proves that the test sample is the closest to the sample category. In addition, the artificial network model was fit using a Gaussian distribution, and the accuracy of the confusion moment discrimination was constructed. The results are shown in Fig 22.

**Fig 23:**
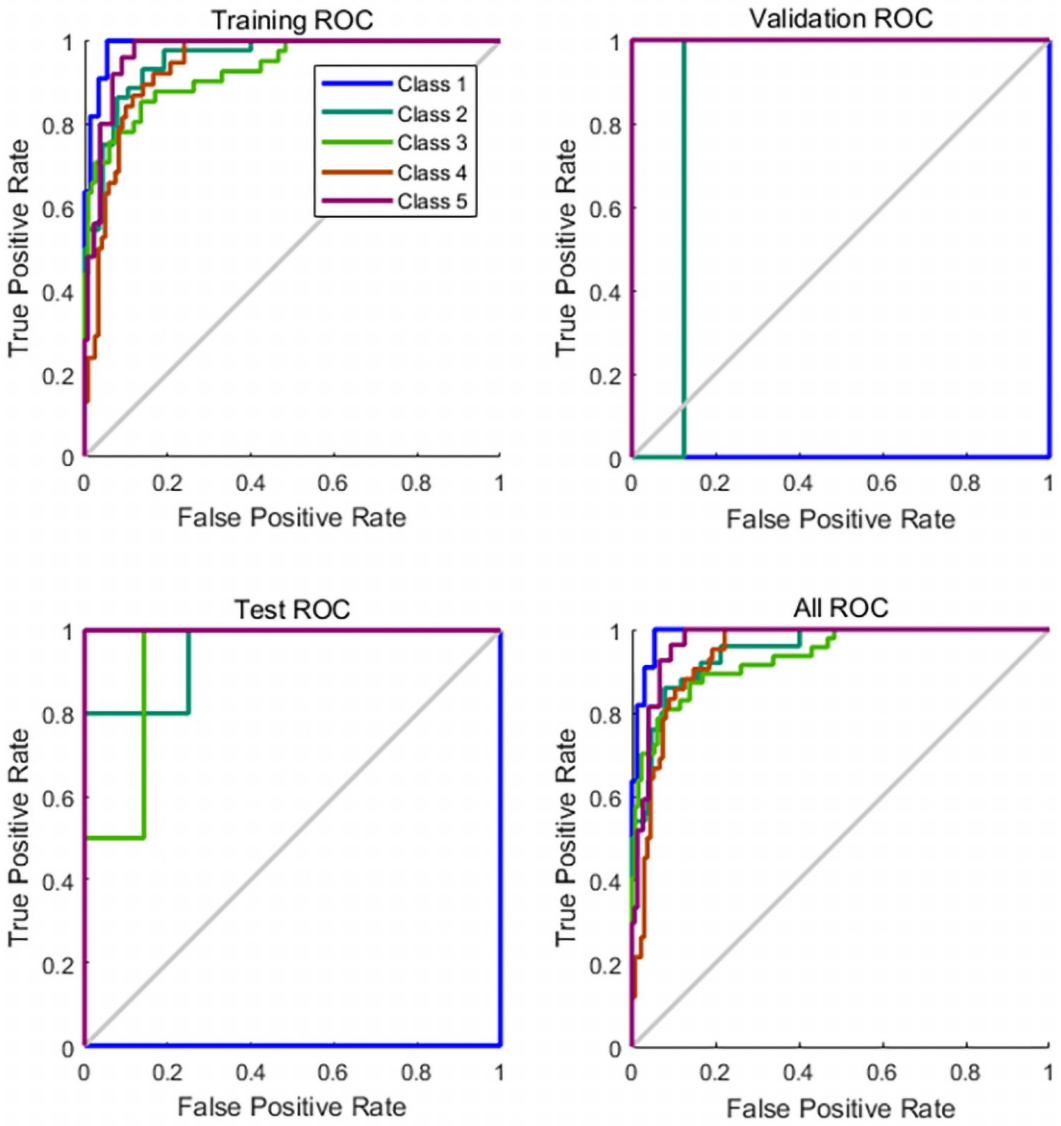
ROC curve diagram.

As shown by the above confusion matrix, the accuracy of the neural network on the test set after training is 88.9%. The training process of five fatigue classifications can be derived from the ROC (Receiver Operating Characteristic) curve, and the ROC results are shown in Fig 23.

Through the ROC curve, the training process of five types of fatigue classification can be obtained. When the curve is closer to 0.1, the correct rate of the training model classification is higher.

### Validation of the classification model

In order to evaluate the driving fatigue of the human body exposed to different vibration conditions, comparative experiments were carried out for these two conditions. We chose a subject to conduct a comparative test under two different vibration conditions. In one set of tests, a subject was exposed to the original vibration on the vibrating platform, and in the other set of tests, we installed a vibration-absorbing suspension on the vibrating platform to reduce the vibration. A quasi-stiffness suspension based on elliptical cam-roller was used to reduce the vibration in this controlled trial. This suspension reduces the shock caused by vibration through the working together of elliptical cam and roller, Gao et al. tested the vibration absorption performance of this suspension in field, the quasi-zero structure maintains the overall stiffness at 0.034 N/m in 20 mm amplitude of field tests. This modified suspension also decreases isolation frequency from 3.87 to 1.67 Hz and quasi-stiffness structure offset 81.3% of positive stiffness [30]. We collected the physiological signals of the subjects and then calculated the physiological parameters. After that, we input these extracted parameters into the previously trained artificial neural network, and compared the changes in fatigue under the two conditions.

**Fig 24:**
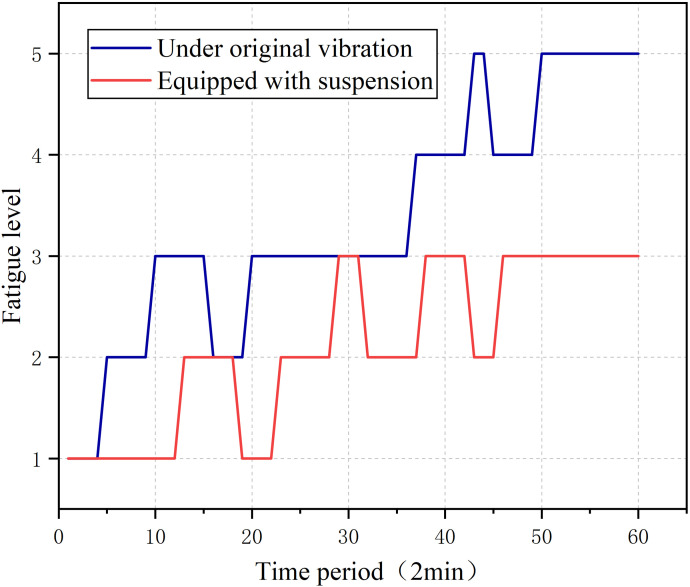
Comparisons and analysis of comfort evaluation between vibration absorbing seat suspension and ordinary seat suspension.

It can be seen from the Fig 24 that the fatigue changes of the subjects under the two different vibration conditions are clearly different. During the 0–15 time period of the test, the fatigue level of the equipped vibration damping suspension is significantly lower than that of exposure to the original vibration. Both fatigue levels decreased significantly during the 15–17 time period, and returned to the previous fatigue level during the 17–20 time period and maintained to the 35 time period. In the later period of the test, the fatigue level of the subjects exposed to the original vibration conditions was significantly higher, and reached the fifth level of fatigue. The difference is that the human body fatigue under the condition of being equipped with a vibration damping suspension does not reach above level three fatigue.

## Conclusion

This article focuses on the evaluation of tractor driving vibration fatigue and proposes a method for evaluating tractor vibration driving fatigue based on multiple physiological parameters. First, we input the vibration collected under natural conditions to the vibration platform to simulate the vibration of the human body when a tractor is driving. Then the four physiological signals collected were preprocessed, and each characteristic value was extracted separately. The results show that with the continuation of driving time, the median frequency value of human surface EMG decreases, the average value of skin electricity increases, the slope of skin temperature decreases, and the average value of photoplethysmography signal increases. In addition, the change of fatigue level was verified by a subjective questionnaire survey.

This article further verifies the fatigue of driving vibration by constructing an artificial neural network. Using the extracted physiological characteristic parameters to train the artificial neural network, the accuracy of the model’s classification of fatigue levels reached 88.9%. This classification model was used to evaluate the changes in human driving fatigue levels under two vibration conditions. Using the sub-model to compare the fatigue levels of the human body under different vibration environments, it was found that the driving fatigue level of installing the vibration-damping suspension was much lower than the fatigue level experienced when exposed to the original vibration. In conclusion, this paper proposes a multiple physiological parameters based vibration fatigue evaluation method for tractor driving. We carried out several experiments to verify the effectiveness of this method. It provides a reference for the follow-up tractor fatigue prediction, human fatigue warning, and the tractor’s vibration damping effect.

## Supporting information

S1 File Four physiological signals. (RAR)

S2 File Relevant information. (DOCX)
