# Supplementary material for: Evaluation of tractor driving vibration fatigue based on multiple physiological parameters
Source: PLoS One. 2021 Jul 14;16(7):e0254636. doi: 10.1371/journal.pone.0254636 (PMC8279742; doi:10.1371/journal.pone.0254636)
Supplement: S2 File — (DOCX) [file pone.0254636.s002.docx]

<http://apps.webofknowledge.com/full_record.do?product=UA&search_mode=GeneralSearch&qid=1&SID=8Bui6epMRypTW7P5Uqs&page=1&doc=7>

<http://apps.webofknowledge.com/CitedFullRecord.do?product=UA&colName=WOS&SID=8Bui6epMRypTW7P5Uqs&search_mode=CitedFullRecord&isickref=WOS:000355243800001>

<http://apps.webofknowledge.com/CitedFullRecord.do?product=UA&colName=WOS&SID=8Bui6epMRypTW7P5Uqs&search_mode=CitedFullRecord&isickref=WOS:000253598100030>

<https://kns.cnki.net/kcms/detail/detail.aspx?dbcode=CDFD&dbname=CDFDLAST2016&filename=1016085321.nh&v=j8fjtqQKrr7E%25mmd2FFgwq8CCAMobU7qVwKyGZQ5tZe%25mmd2ByP7loPjcxTwWDuNuUPgCIsnu%25mmd2F>
